# Applying a computer model to evaluate the evolution of resistance by western corn rootworm to multiple Bt traits in transgenic maize

**DOI:** 10.1093/jee/toae260

**Published:** 2024-11-05

**Authors:** John B McCulloch, Aaron J Gassmann

**Affiliations:** Department of Plant Pathology, Entomology and Microbiology, Iowa State University, Ames, IA, USA; Department of Plant Pathology, Entomology and Microbiology, Iowa State University, Ames, IA, USA

**Keywords:** *Bacillus thuringiensis*, fitness cost, inheritance of resistance, resistance allele frequency, pyramid/refuge strategy

## Abstract

Western corn rootworm, *Diabrotica virgifera virgifera* (LeConte) (Coleoptera: Chrysomelidae), is a major pest of maize in the United States. Transgenic maize producing insecticidal toxins from the bacterium *Bacillus thuringiensis* (Bt) have been used to manage this pest since 2003. Refuges of non-Bt maize have been used to delay resistance to Bt maize by western corn rootworm, and are planted in conjunction with maize producing single or multiple (i.e., pyramids) Bt toxins. Two Bt toxins, Cry3Bb1 and Gpp34/Tpp35Ab1, were used individually before being combined as a pyramid, at which point resistance had already evolved to Cry3Bb1. Pyramids targeting western corn rootworm therefore contained at least one toxin to which resistance had evolved. Western corn rootworm has now evolved resistance to all four commercially available Bt toxins used for rootworm management. We used laboratory and field-generated data to parameterize a deterministic model to simulate the effectiveness of refuges and Bt pyramids to delay resistance to Bt maize in western corn rootworm. Resistance to the pyramid of Cry3Bb1 with Gpp34/Tpp35Ab1 evolved more rapidly when resistance to Cry3Bb1 was already present. This effect arose when model conditions affecting initial resistance allele frequency, inheritance of resistance, and fitness costs were varied. Generally, resistance evolved faster when initial resistance allele frequencies were higher, inheritance of resistance was nonrecessive, and fitness costs were absent, which is consistent with previous models that simulated resistance evolution. We conclude that new transgenic pyramids should pair novel, independently acting toxins with abundant refuges to minimize the risk of rapid resistance evolution.

## Introduction

Western corn rootworm, *Diabrotica virgifera virgifera* (LeConte) (Coleoptera: Chrysomelidae), is currently the most serious insect pest of maize in the central United States. Management costs and yield losses from western corn rootworm can cost US farmers up to $2 billion per year (Wechsler and Smith 2018). This insect has one generation per year and although larvae are able to successfully develop on a limited number of hosts in the Poaceae, maize is the only agricultural crop on which this pest can survive (Branson and Ortman 1967, 1970, Clark and Hibbard 2004, Moeser and Hibbard 2005, Meinke et al. 2009). Because the scale of monoculture maize in the Midwest is so large, hosts besides maize are typically not considered significant for population dynamics of western corn rootworm. Females are attracted to maize fields as oviposition sites, ensuring the next generation has suitable hosts upon which to feed and develop if fields are not rotated to a non-maize crop (Branson and Krysan 1981). The larvae feed on developing maize roots, reducing water and nutrient uptake (Kahler et al. 1985). Western corn rootworm has been managed using crop rotation, insecticides, and more recently, transgenic maize producing toxins from the bacterium *Bacillus thuringiensis* (Bt) (Gillette 1912, Levine and Oloumi-Sadeghi 1991, Wechsler and Smith 2018, Meinke et al. 2021). Resistance to these management tactics has been documented in various parts of the maize growing region of the United States, presenting challenges for the management of this pest (Levine et al. 2002, Kiss et al. 2005, Spencer et al. 2005, Gassmann et al. 2020, Meinke et al. 2021).

There are four Bt toxins available for management of western corn rootworm in transgenic maize: Cry3Bb1, eCry3.1Ab, Gpp34/Tpp35Ab1, and mCry3A (EPA 2020). With the exception of eCry3.1Ab, these Bt traits were released singly and were later combined in maize hybrids that produced two Bt toxins (i.e., a pyramid) (EPA 2015). The first Bt trait released for management of western corn rootworm was Cry3Bb1, which was made available to farmers in 2003. By 2009, field-evolved resistance to Cry3Bb1 maize was identified for western corn rootworm populations in Iowa (Gassmann et al. 2011). Research in other states also identified resistance to Cry3Bb1 maize in the western Corn Belt (Wangila et al. 2015, Zukoff et al. 2016, Schrader et al. 2017, Reinders et al. 2018, Calles-Torrez et al. 2019). Subsequent studies found cross-resistance among Cry3Bb1, eCry3.1Ab, and mCry3A (Gassmann et al. 2014, Wangila et al. 2015, Jakka et al. 2016, Zukoff et al. 2016). Beginning in 2013, evidence of field-evolved resistance to Gpp34/Tpp35Ab1 also was found in the western Corn Belt (Gassmann et al. 2014, Ludwick et al. 2017, Reinders and Meinke 2022, Reinders et al. 2022). In general, populations with resistance to Gpp34/Tpp35Ab1 were also resistant to Cry3Bb1, and due to cross-resistance among Cry3 traits, also mCry3A and eCry3.1Ab.

The United States Environmental Protection Agency (US EPA) mandates a resistance management plan for the commercial cultivation of all transgenic insecticidal crops (EPA 2023). Resistance management strategies used in the United States focus on the refuge strategy, where non-Bt host plants are planted near, or within, fields with Bt crops (Gould 1998, EPA 2023). Refuges are also used with pyramid Bt crops. The pyramid/refuge strategy delays resistance because most alleles for resistance to one toxin in a pyramid reside within individuals that are susceptible to the other toxin. As a result, pyramids remove resistance alleles from the population by killing individuals that are susceptible to one of the toxins (Gould 1998, Roush 1998, Andow 2008). In the case of the pyramid/refuge strategy, non-Bt refuges allow the survival of susceptible individuals that can mate with resistant individuals reducing the proportion of individuals that harbor traits for resistance to both toxins in a pyramid (Roush 1997, 1998, Gould 1998, Andow 2008).

Several factors can influence the effectiveness of the refuge strategy to delay resistance including: inheritance of resistance, initial resistance allele frequency, and fitness costs (Taylor and Georghiou 1979, Gould et al. 1997, Roush 1998, Tabashnik et al. 2004, Andow 2008, Brévault et al. 2013). The risk of resistance evolution increases as the inheritance of resistance traits become more dominant, and larger refuges are required to delay resistance if the inheritance of resistance is nonrecessive compared to cases where resistance is recessive (Tabashnik et al. 2008, 2023b, Jin et al. 2015). Fitness costs of resistance occur on non-Bt hosts when resistant individuals have lower fitness than susceptible individuals, and fitness costs select against resistance in refuges (Gassmann et al. 2009a). The capacity of the pyramid/refuge strategy to delay resistance is affected by the frequency of alleles for resistance to either Bt toxin in a pyramid (Roush 1998, Tabashnik and Gould 2012). In particular, an increase in the frequency of resistance alleles for either toxin in a Bt pyramid can diminish the capacity of the pyramid to delay resistance (Roush 1998, Tabashnik and Gould 2012).

One of the primary challenges with managing resistance to Bt maize by western corn rootworm has been the use of, and evolution of resistance to, Bt traits singly prior to combining them in a Bt pyramid. The evolution of resistance to single-trait Bt crops facilitates evolution of resistance to pyramids which include those Bt traits (Gassmann 2021, Gassmann and Reisig 2023, Gassmann and Meinke 2024). In particular, resistance to Cry3Bb1 maize by western corn rootworm reduced the capacity of maize with a pyramid of Cry3Bb1 and Gpp34/Tpp35Ab1 to delay resistance, and western corn rootworm subsequently evolved resistance to maize with a pyramid of Cry3Bb1 and Gpp34/Tpp35Ab1 (Gassmann 2021). In Iowa, where Cry3Bb1 resistance was identified initially in 2009, a subsequent field study found that resistance to Cry3Bb1in western corn rootworm was pervasive within the state by 2014 (Shrestha et al. 2018). Similar results were found for western corn rootworm populations in Nebraska (Reinders et al. 2022). One way that farmers managed resistance to Cry3 toxins was by planting maize with a pyramid of Cry3Bb1 and Gpp34/Tpp35Ab1 (Dunbar et al. 2016, Shrestha et al. 2018, Gassmann 2021, Gassmann and Reisig 2023, Gassmann and Meinke 2024). This in turn led to the evolution of populations with resistance to this Bt pyramid (Gassmann et al. 2020, Gassmann 2021, Reinders et al. 2022).

In this study, we used data from laboratory and field studies to model the evolution of resistance by western corn rootworm to maize producing Cry3Bb1 and Gpp34/Tpp35Ab1, both as single-toxin and pyramid cultivars. The results of this model illustrate factors facilitating the evolution of resistance to Bt maize by western corn rootworm and approaches for improving resistance management for current and future transgenic traits.

## Materials and Methods

Although there is evidence to suggest that resistance to Bt toxins by western corn rootworm involves a differential midgut epithelial response and may be oligogenic or polygenic, the underlying genetic mechanisms of resistance are unknown (Carrière et al. 2010, Flagel et al. 2015, Thompson 2014, Gassmann 2016, Paddock et al. 2023). In such cases, monogenic genetic models are typically assumed and are expected to produce more conservative results (Gassmann et al. 2009b, Onstad and Knolhoff 2023). We therefore modeled evolution of resistance to Bt maize by western corn rootworm using a model with two loci, and two alleles at each locus. All individuals were diploid. One locus coded for resistance to Cry3Bb1 and had alleles for resistance (R_Cry3Bb1_) or susceptibility (S_Cry3Bb1_). The other locus coded for resistance to Gpp34/Tpp35Ab1 and was similarly structured with two alleles (R_Gpp34/Tpp35Ab1_ or S_Gpp34/Tpp35Ab1_).

**Fig. 1.**
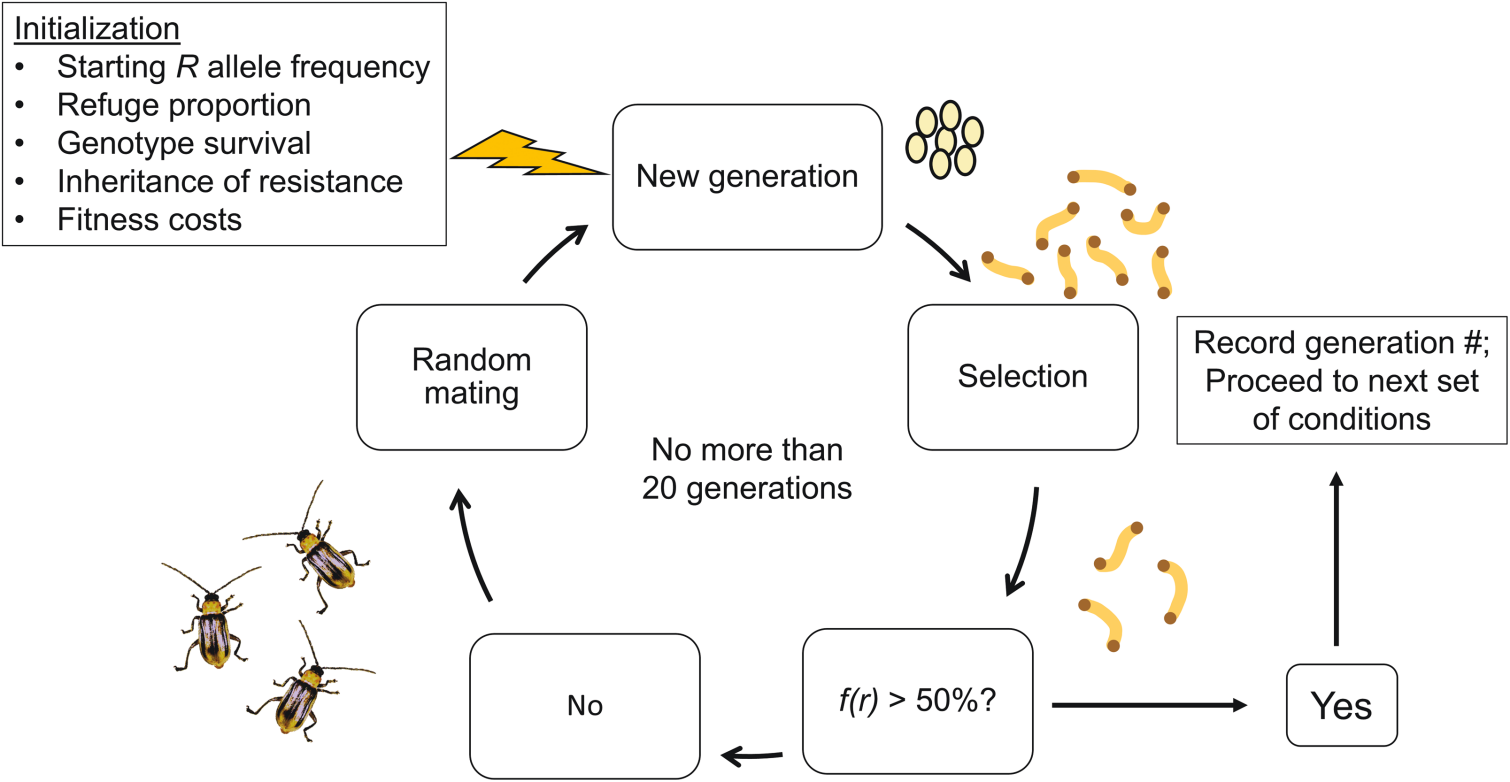
Visual model for assessing time to resistance of western corn rootworm (*Diabrotica virgifera virgifera*) on Bt maize producing Cry3Bb1 and Gpp34/Tpp35Ab1, either individually or together in a pyramid. A simulation is initialized by a set of input parameters, generating a population of eggs. The eggs hatch and larvae undergo selection on Bt and non-Bt maize. Surviving larvae develop to adults and mate randomly to produce the next generation. For simulations of single-toxin maize, only the resistant allele frequency (*f*(*r*)) at the locus under selection is assessed for 50% frequency. In simulations with pyramid maize, the resistant allele frequency at both loci are assessed for 50% frequency. Time to resistance was assessed for simulations that varied in proportion non-Bt refuge, inheritance of resistance, initial resistance allele frequency, fitness costs of resistance, and previous resistance to Cry3Bb1 (when evaluating pyramid maize producing both Cry3Bb1 and Gpp34/Tpp35Ab1).

The model was constructed in Python 3 using the Jupyter Notebook interface and followed the general format of Roush (1998) (Supplementary Appendix 1). Many of the assumptions listed in Roush (1998) were also used in this model, including space is implicit, each time step is one generation, mortality factors besides Bt selection are negligible, density-dependent larval mortality is absent, larvae do not move (i.e., they only feed on one host plant for their entire development), and surviving adults mate randomly. Past research has found that limited adult movement and protandry can substantially diminish random mating between western corn rootworm adults emerging from refuge plants versus Bt plants when refuges are spatially distinct from Bt fields within the landscape (Pan et al. 2011, Spencer et al. 2013, Hughson and Spencer 2015). When refuge plants and Bt plants are interspersed, as is the case with a blended or integrated refuge, nonrandom mating is likely less pronounced. As such, our assumption of random mating more accurately captures the dynamic of an integrated refuge compared to a spatially structured refuge (Crowder and Onstad 2005, Onstad 2006). To evaluate the model for proper functionality, we verified that allele frequencies did not change across time when both selection for Bt resistance and fitness costs of resistance were removed, and, using our own code, we reproduced Fig. 1 from Roush (1998) based on the same parameter values (Supplementary Appendix 2). A visual representation of the model is presented in Fig. 1.

We evaluated the time to resistance across refuge proportions from 0 to 1, in 0.01 increments for single-trait Cry3Bb1 maize, single-trait Gpp34/Tpp35Ab1, and a pyramid of the two, assuming no previous selection from Bt maize (Supplementary Appendices 3 and 4). We also evaluated time to resistance for a Bt pyramid of Cry3Bb1 and Gpp34/Tpp35Ab1 when resistance to Cry3Bb1 was already present. A population underwent selection for Bt resistance at the larval stage and surviving individuals mated randomly. In the case of maize with a single Bt toxin, a population was defined as evolving resistance when the resistance allele frequency reached 50%. For simulations of maize with a Bt pyramid, the resistance allele frequency for both toxins needed to reach 50%. A simulation was ended if a resistance allele frequency remained below 50% after 20 generations.

Previously published studies were used to obtain baseline values of each parameter. For survival on Bt hybrids, studies were included that provided data on survival to adulthood, of Bt-susceptible western corn rootworm in the field, on pure stands of both Bt and non-Bt maize. For inheritance of resistance, we used studies that provided data on survival to adulthood, on Bt and non-Bt maize, for Bt-susceptible, Bt-resistant, and heterozygous (resistant × susceptible crosses) western corn rootworm strains with a similar genetic background. For fitness costs of resistance, studies were included that provided data on at least survival to adulthood on non-Bt maize for resistant and susceptible western corn rootworm with a similar genetic background. However, data on other life-history metrics such as fecundity, egg viability, etc., were also included in our calculation of fitness costs if those data were reported in a study. The value of the common assumption for initial resistance allele frequency, often used when empirical data is absent, was set at 0.001 based on Roush (1998), while Onstad and Meinke (2010) provide the only data available for realistic values of initial resistance allele frequencies to Cry3Bb1 and Gpp34/Tpp35Ab1 in western corn rootworm.

Baseline values for survival for Bt-susceptible individuals on Bt maize, inheritance of resistance, and fitness costs of resistance, for each Bt toxin, were calculated as the average of the values presented in the literature (Table 1). Proportion survival of RR individuals on Bt maize (*W*_RR_) and survival of individuals for all genotypes on non-Bt maize were set at 1. Survival of Bt-susceptible individuals on Bt maize (*W*_SS_) were based on values reported in the peer-reviewed literature for field studies. Where a single study tested a susceptible population across multiple years and field locations, each combination of year by location was treated as an independent observation, which included data from Hitchon et al. (2015) and Shrestha and Gassmann (2019). Where various methods of calculating survival were used (e.g., with or without density-dependent mortality), the average value among the methods was used, which included data from Shrestha et al. (2016). If mortality was calculated for different treatments in Bt maize (e.g., insecticidal seed coatings or different egg densities) and these factors did not significantly affect survival compared to Bt maize alone (e.g., insecticide seed coating), the value for each treatment was considered an independent observation, which included data from Petzold-Maxwell et al. (2013b) and Hitchon et al. (2015). Based on our review of the literature, survival of susceptible individuals (*W*_SS_) was set to 0.104 on Cry3Bb1 maize and 0.118 on Gpp34/Tpp35Ab1 maize (Table 1). For simulations evaluating the pyramid of Cry3Bb1 and Gpp34/Tpp35Ab1, survival on the pyramid for Bt-susceptible individuals was multiplicative, and calculated as the product of survival on single-toxin Cry3Bb1 and Gpp34/Tpp35Ab1 for each genotype, with a resulting value of 0.012 (i.e., 0.104 × 0.118) (Supplementary Table 1). We assumed that mortality imposed by each toxin in the pyramid acted in a multiplicative manner. However, as shown by Head et al. (2014), the effect of Cry3Bb1 and Gpp34/Tpp35Ab1 on western corn rootworm may be less than multiplicative, and future modeling work could explore how altering the interactive effects of Bt toxins on mortality of western corn rootworm affects the dynamics of resistance evolution.

**Table 1.** Values and sources for model parameters for western corn rootworm (*Diabrotica virgifera virgifera*) on Bt maize producing insecticidal toxins Cry3Bb1 or Gpp34/Tpp35Ab1^a^

Parameter^b^	Average	Range	Source
**Cry3Bb1**			
Survival	0.23	0.21–0.25	Petzold-Maxwell et al. (2013b)
Survival	0.048	0.033–0.078	Hitchon et al. (2015)
Survival	0.395	na	Shrestha et al. (2016)
Survival	0.029	0.019–0.039	Shrestha and Gassmann (2019)
Mean survival	0.104	0.019–0.395	**–**
Inheritance of resistance	0.32	0.27–0.37	Ingber and Gassmann (2015)
Inheritance of resistance	0.40	0.14–0.73	Paolino and Gassmann (2017)
Mean inheritance of resistance	0.375	0.14–0.73	**–**
Cry3Bb1 fitness costs	7.5%	0–15%	Ingber and Gassmann (2015)
Cry3Bb1 fitness costs	0.093%	0–0.19%	Paolino and Gassmann (2017)
Cry3Bb1 fitness costs	4.39%	3.18–5.34%	St. Clair et al. (2020)
Mean Cry3Bb1 fitness costs	4.10%	0–15%	**–**
Common initial R allele frequency	0.001	na	Roush (1998)
Realistic initial R allele frequency	0.2	na	Onstad and Meinke (2010)
**Gpp34/Tpp35Ab1**			
Survival	0.143	0.109–0.177	Petzold-Maxwell et al. (2013a)
Survival	0.131	0.030–0.337	Hitchon et al. (2015)
Survival	0.19	na	Shrestha et al. (2016)
Survival	0.012	0.018–0.006	Shrestha and Gassmann (2019)
Mean survival	0.118	0.018–0.337	**–**
Inheritance of resistance	0.36	na	Smith et al. (2023)
Fitness costs	5.55%	na	Smith et al. (2023)
Common initial R allele frequency	0.001	na	Roush (1998)
Realistic initial R allele frequency	0.075	0.05–0.1	Onstad and Meinke (2010)

^a^Averages and ranges are given where multiple data points were presented in one source. If only one value was present in a source, that value is given as the average and range is not applicable (na).

^b^Values for survival, in field settings, are for susceptible individuals on Bt maize compared to susceptible individuals on non-Bt maize (ranges from 0 to 1). Inheritance of resistance, the difference in survival of heterozygous individuals from homozygous susceptible individuals (on Bt maize) compared to that for homozygous resistant individuals, were generated in laboratory bioassays (ranges from 0 to 1). Fitness costs, the difference in fitness metrics of resistant individuals compared to susceptible individuals on non-Bt maize, were also generated in laboratory bioassays (ranges from 0% to 100%). Researchers often assume the common initial R allele frequency when no empirical data exist; the realistic initial R allele frequency was estimated by Onstad and Meinke (2010) for western corn rootworm on Bt maize producing either Cry3Bb1 or Gpp34/Tpp35Ab1 using population models fit to previously published survival data (Meihls et al. (2008) for Cry3Bb1 and, Lefko et al. (2008) for Gpp34/Tpp35Ab1).

Survival of heterozygous individuals (*W*_RS_) on single-toxin Bt maize was calculated based on the formula *h* = (*W*_RS_ − *W*_SS_)/(*W*_RR_ − *W*_SS_), with *h* (inheritance of resistance) determined based on values in the peer-reviewed literature (Table 1) and *W*_SS_ and *W*_RR_ determined as previously described. Inheritance of resistance for Cry3Bb1 and Gpp34/Tpp35Ab1 was calculated as the average of the values presented in the literature. Where multiple strains of western corn rootworm, or different bioassays for the same strain, were used to test inheritance of resistance within a single study, the values were treated as independent observations. Unless otherwise noted, the values for inheritance of resistance used in the model were 0.375 for Cry3Bb1 and 0.36 for Gpp34/Tpp35Ab1 (Table 1), which translates to *W*_RS_ = 0.440 for Cry3Bb1 resistance and *W*_RS_ = 0.436 for Gpp34/Tpp35Ab1 resistance.

Survival on non-Bt maize for homozygous resistant individuals was either set at 1 if no fitness costs were present or less than 1 to account for fitness costs. In the model, fitness costs were defined as lower larval survival for individuals with resistance alleles on non-Bt maize compared to homozygous susceptible individuals. We assumed that fitness costs translated directly to reduced survival on non-Bt maize. Survival on Bt maize was not affected by fitness costs. When reviewing the literature to determine the value for fitness costs, we averaged values if multiple parameters provided evidence of a cost. The average fitness cost of 4.1% was used for Cry3Bb1 resistance and a 5.55% cost was used for Gpp34/Tpp35Ab1 resistance (Table 1), and these costs were considered recessive, meaning that survival costs did not affect survival on non-Bt maize for individuals heterozygous for resistance. To evaluate the impact of lower and higher fitness costs, we used the lower (0%) and upper (15%) values for fitness costs of Cry3Bb1 resistance presented in the literature. These same values were used for resistance for Gpp34/Tpp35Ab1 since only one study, Smith et al. (2023), has estimated fitness costs for various life-history parameters on Bt maize producing Gpp34/Tpp35Ab1. The higher value for fitness costs also included a nonrecessive cost, with heterozygotes experiencing 0.4 times the fitness costs experienced by resistant individuals (i.e., 6%: 15% × 0.4) based on the literature review presented in Gassmann et al. (2009).

The model evaluated a total of six scenarios. The first scenario used initial resistance allele frequencies based on the commonly assumed value of 0.001 (Roush 1998). The second scenario used more realistic values for western corn rootworm of 0.2 for Cry3Bb1 and 0.075 for Gpp34/Tpp35Ab1 (Table 1) (Lefko et al. 2008, Meihls et al. 2008, Onstad and Meinke 2010). The third and fourth scenarios varied inheritance of resistance and the fifth and six scenarios varied fitness costs of resistance (Supplementary Table 1).

**Fig. 2.**
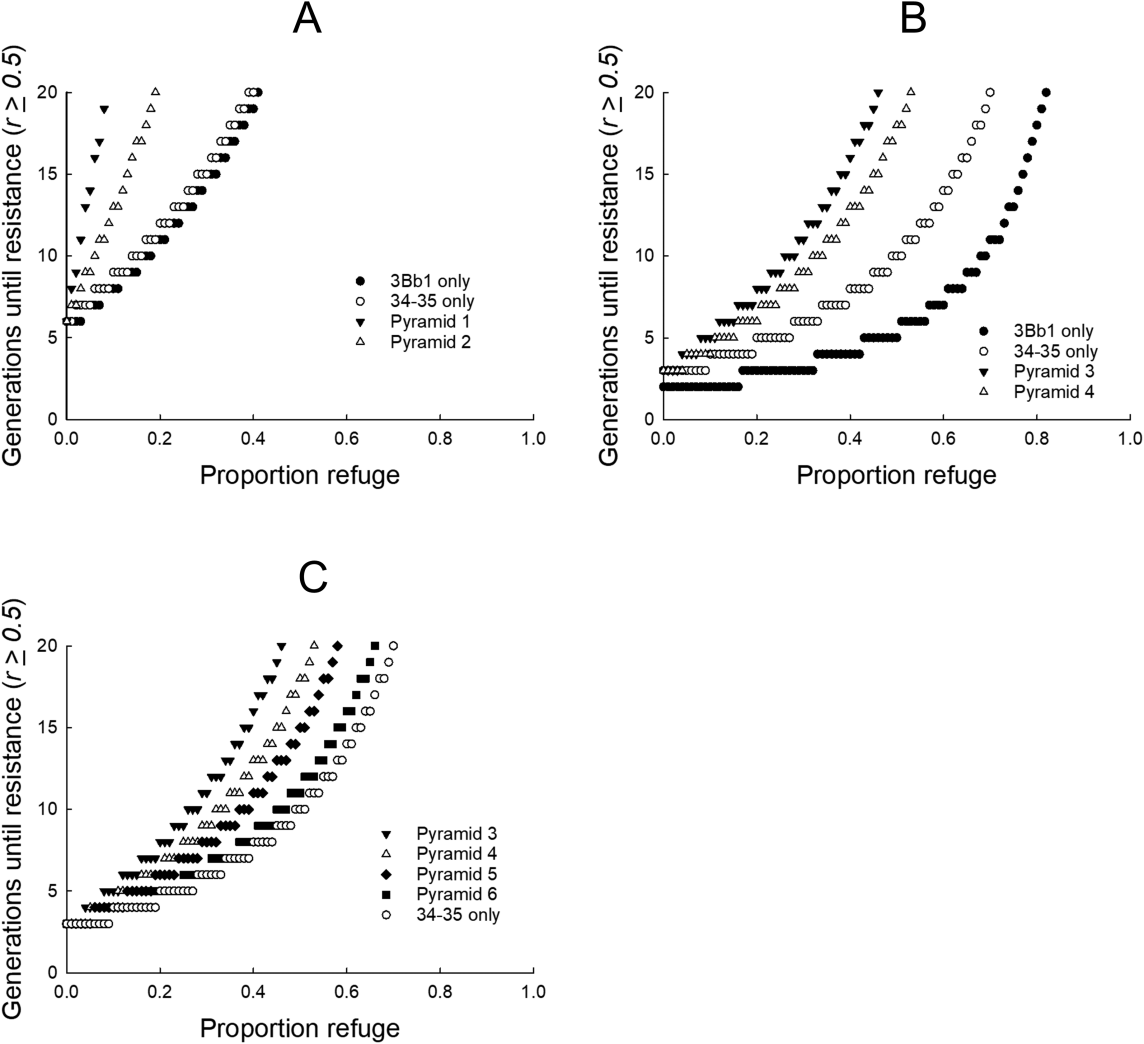
Effects of initial resistance (*R*) allele frequency and Bt pyramids (*Pyr*) on time to resistance. Resistance was defined as occurring when the *R* allele frequency ≥0.50 for single-toxin maize or ≥0.50 for both toxins in *Pyr*. Baseline values for inheritance of *R* (0.375 for Cry3Bb1 and 0.36 for Gpp34/Tpp35Ab1) and fitness costs (4.1% for Cry3Bb1 and 5.55% for Gpp34/Tpp35Ab1) were used in all simulations (Table 1). A) *Pyr* 1 used initial *R* allele frequency of 0.001 for both toxins while *Pyr* 2 used a frequency of 0.1 for Cry3Bb1 and 0.001 for Gpp34/Tpp35Ab1. B) *Pyr* 3 used initial *R* allele frequencies of 0.2 for Cry3Bb1 and 0.075 for Gpp34/Tpp35Ab1. *Pyr* 4 used initial *R* allele frequencies of 0.35 for Cry3Bb1 and 0.075 for Gpp34/Tpp35Ab1. C) Initial *R* allele frequency was 0.075 for Gpp34/Tpp35Ab1 maize in all simulations. Initial *R* allele frequency for Cry3Bb1 *R* was *Pyr* 3 = 0.2, *Pyr* 4 = 0.35, *Pyr* 5 = 0.5, and *Pyr* 6 = 0.8.

The first scenario (Fig. 2A) used the baseline (i.e., average) values for inheritance of resistance and fitness costs parameters as derived from the literature (Table 1). *W*_RS_ was 0.440 for Cry3Bb1 resistance and 0.436 for Gpp34/Tpp35Ab1 resistance, fitness costs were 4.1% and 5.55% for resistance to Cry3Bb1 and Gpp34/Tpp35Ab1, respectively. The common assumption of 0.001 was used for the initial resistance allele frequency for resistance to Cry3Bb1 and Gpp34/Tpp45Ab1. For the simulation of the Bt pyramid when Cry3Bb1 resistance was already present, the initial resistant allele frequency for Cry3Bb1 was increased to 0.1 while the initial resistance allele frequency for Gpp34/Tpp35Ab1 remained at 0.001. A resistance allele frequency of 0.1 has been used in past simulation studies to represent resistance to one toxin in a pyramid (Onstad and Meinke 2010, Martinez and Caprio 2016).

**Fig. 3.**
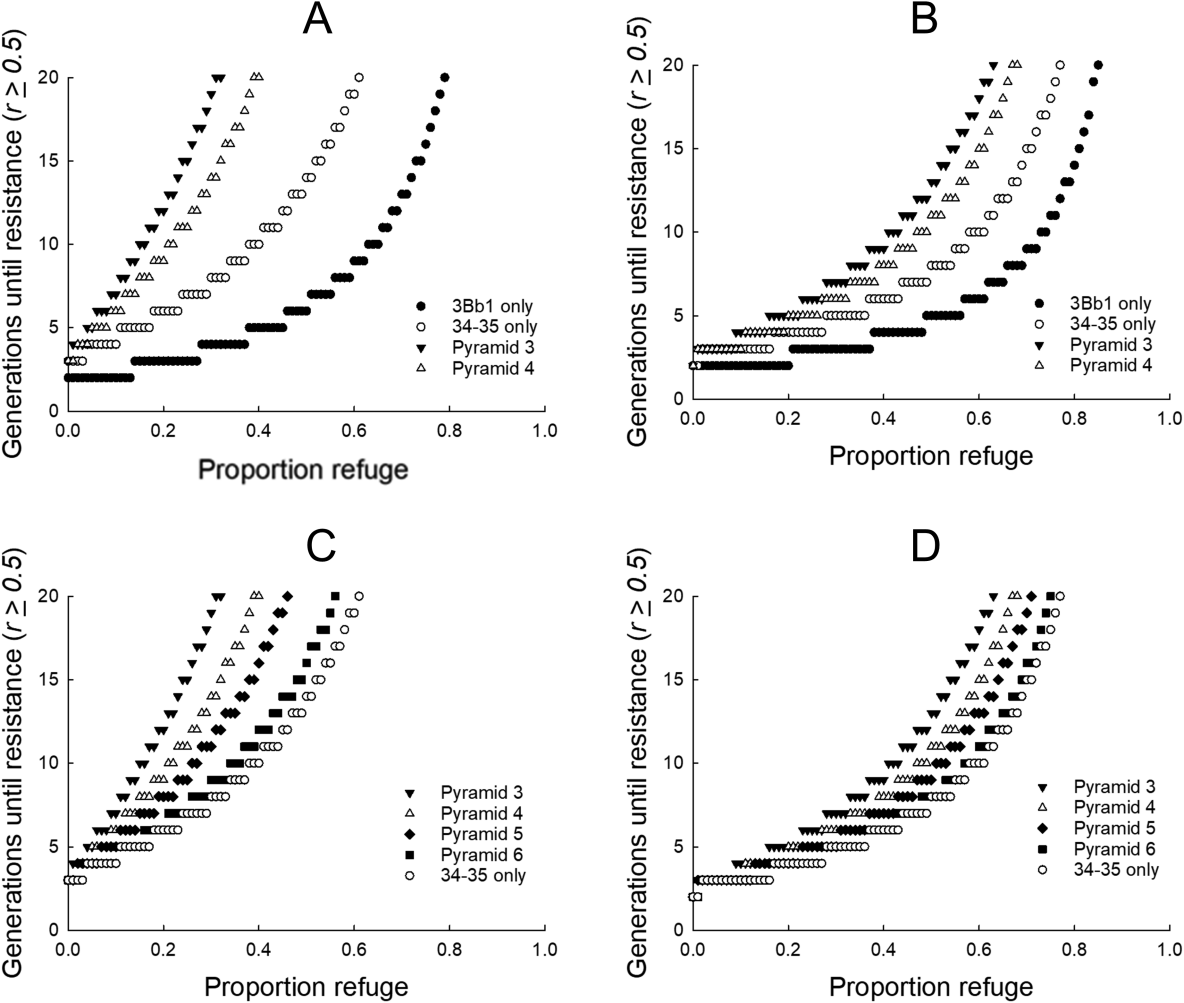
Effects of inheritance of resistance (*R*) traits on rate of resistance evolution. Resistance was defined as occurring when the *R* allele frequency ≥0.50 for single-toxin maize or ≥0.50 for both toxins in pyramids (*Pyr*). Baseline values for fitness costs were used in all simulations: 4.1% for Cry3Bb1 and 5.55% for Gpp34/Tpp35Ab1 (Table 1). A) Inheritance of *R* was set at the lower value of 0.14 for both toxins. Single-toxin maize used initial *R* allele frequencies of 0.2 for Cry3Bb1 and 0.075 for Gpp34/Tpp35Ab1. *Pyr* 3 and *Pyr* 4 used initial *R* allele frequency of 0.075 for Gpp34/Tpp35Ab1. *Pyr* 3 used 0.2 for the initial *R* allele frequency of Cry3Bb1 and *Pyr* 4 used 0.35. B) Inheritance of *R* was set at the higher value of 0.73 for both toxins. Simulations used the same initial *R* allele frequencies as listed in (A). C) Inheritance of *R* was set to the lower value of 0.14 for both toxins. Initial *R* allele frequency was 0.075 for Gpp34/Tpp35Ab1 maize in all simulations. Initial *R* allele frequency for Cry3Bb1 *R* was *Pyr* 3 = 0.2, *Pyr* 4 = 0.35, *Pyr* 5 = 0.5, and *Pyr* 6 = 0.8. D) Inheritance of *R* was set to the higher value of 0.73 for both toxins. Simulations used the same initial *R* allele frequencies as listed in (C).

**Fig. 4.**
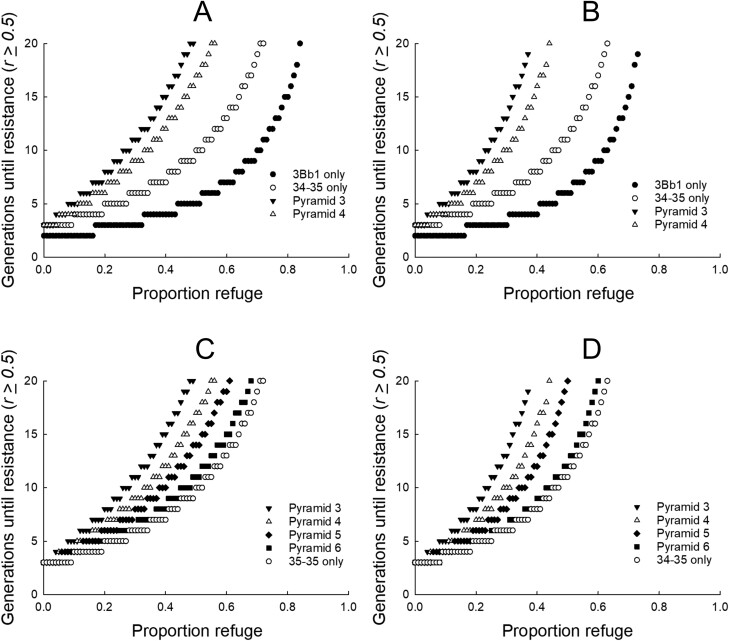
Effect of fitness costs on the rate of resistance (*R*) evolution. Resistance was defined as occurring when the *R* allele frequency ≥0.50 for single-toxin maize or ≥0.50 for both toxins in pyramids (*Pyr*). Baseline values for inheritance of *R* were used in all simulations: 0.375 for Cry3Bb1 and 0.36 for Gpp34/Tpp35Ab1 (Table 1). A) Fitness costs for both Cry3BB1 and Gpp34/Tpp35Ab1 were absent. Single-toxin maize used initial *R* allele frequencies of 0.2 for Cry3Bb1 and 0.075 for Gpp34/Tpp35Ab1. *Pyr* 3 and *Pyr* 4 used an initial *R* allele frequency of 0.075 for Gpp34/Tpp35Ab1. *Pyr* 3 used 0.2 for the initial *R* allele frequency of Cry3Bb1 and *Pyr* 4 used 0.35. B) Fitness costs were higher and nonrecessive: 15% for homozygous resistant individuals and 6% for heterozygous individuals. Simulations used the same initial *R* allele frequencies as listed in (A). C) Fitness costs were absent for both Cry3Bb1 and Gpp34/Tpp35Ab1. Initial *R* allele frequency was 0.075 for Gpp34/Tpp35Ab1 maize in all simulations. Initial *R* allele frequency for Cry3Bb1 *R* was *Pyr* 3 = 0.2, *Pyr* 4 = 0.35, *Pyr* 5 = 0.5, and *Pyr* 6 = 0.8. D) Fitness costs were higher and nonrecessive: 15% for homozygous *R* individuals and 6% for heterozygous individuals. Simulations used the same initial *R* allele frequencies as listed in (C).

The second scenario (Fig. 2B) used more realistic values for initial resistance allele frequencies for Cry3Bb1 resistance (0.2) and Gpp34/Tpp35Ab1 resistance (0.075), along with baseline values for inheritance of resistance and fitness costs (Table 1). In the simulation of the pyramid when Cry3Bb1 resistance was already present, the initial resistant allele frequency was set at 0.35 for Cry3Bb1 resistance, which is halfway between the realistic initial frequency for Cry3Bb1 and the cutoff for resistance evolution (i.e., 0.50), while initial resistance allele frequency for Gpp34/Tpp35Ab1 remained at 0.075. The values for initial resistant allele frequency described for this scenario (Fig. 2B) were used in all subsequent scenarios unless otherwise noted (Figs. 3 and 4).

The third and fourth scenarios evaluated time to resistance using the upper and lower bounds for the inheritance of resistance. Thompson (2014) used sublethal bioassays to determine that inheritance of resistance to Gpp34/Tpp35Ab1 could range from 0.28 to 0.86. However, because these data did not use survival to adulthood, we omitted these data. Because only one study was found that estimated the inheritance of resistance using survival to adulthood for Gpp34/Tpp35Ab1, we used the lower (0.14) and upper (0.73) values of Cry3Bb1 for both Cry3Bb1 and Gpp34/Tpp35Ab1 when evaluating the impact of low and high values for inheritance of resistance in the model (Fig. 3; Smith et al. 2023). An inheritance value of 0.14 translated to *W*_RS_ = 0.229 and 0.241 for Cry3Bb1 and Gpp34/Tpp35Ab1, respectively; an inheritance value of 0.73 translated to *W*_RS_ = 0.758 and 0.762 for Cry3Bb1 and Tpp34/Tpp35Ab1, respectively. The third scenario (Fig. 3A and C) used baseline values for fitness costs of resistance and used the lower bound of 0.14 for the inheritance of resistance. The fourth scenario (Fig. 3B and D) used baseline values for fitness costs of resistance and used the upper bound of 0.73 for the inheritance of resistance.

The fifth and sixth scenarios evaluated time to resistance for upper and lower bounds of fitness costs. The fifth scenario (Fig. 4A and C) used no fitness costs at either locus, with survival of all genotypes on non-Bt maize equal to 1. The sixth scenario (Fig. 4B and D) used nonrecessive fitness costs, where homozygous resistant individuals experienced 15% reduced larval survival and heterozygous individuals experienced 6% reduced larval survival on non-Bt maize.

We also explored time to resistance in scenarios 2–6 for a pyramid of Cry3Bb1 with Gpp34/Tpp35Ab1 for different levels of previous resistance to Cry3Bb1. The previously described parameter values for each scenario were used except for the initial frequency for Cry3Bb1-resistance alleles. We simulated previous resistance to Cry3Bb1 by running separate simulations using initial allele frequencies for Cry3Bb1 resistance of 0.2, 0.35, 0.5, and 0.8, while the initial resistant frequency for Gpp34/Tpp35Ab1 was always set at 0.075. Because 0.2 is the realistic initial resistant allele frequency for Cry3Bb1, simulations using this value represent no previous exposure to Cry3Bb1 when the pyramid became commercially available. Also, the respective simulations of single-toxin Gpp34/Tpp35Ab1 were included for each scenario as a reference of comparison.

## Results

We found that time to resistance increased rapidly (i.e., resistance was delayed) as refuge size increased for single-toxin and pyramid Bt maize when the common assumption of 0.001 was used as an initial resistance allele frequency for Cry3Bb1 and Gpp34/Tpp35Ab1 (Fig. 2A). Single-toxin Cry3Bb1 and Gpp34/Tpp35Ab1 performed similarly, and time to resistance increased from 6 generations to 20 generations as refuge proportion increased from 0 to 0.4 (Fig. 2A). The greatest delays in resistance were achieved for the pyramid without previous resistance to Cry3Bb1, while delays in resistance for the pyramid with previous Cry3Bb1 resistance (i.e., with an initial resistance allele frequency of 0.1) were intermediate between a single Bt trait and a pyramid without previous resistance (Fig. 2A).

Time to resistance was reduced in the second scenario when more realistic initial resistance allele frequencies were used (0.2 for Cry3Bb1 and 0.075 for Gpp34/Tpp35Ab1) compared to 0.001 for both toxins in the first scenario (Fig. 2A and B). Time to resistance was nearly doubled for single-toxin Gpp34/Tpp35Ab1 maize compared to single-toxin Cry3Bb1 maize (Fig. 2B). Maize with a pyramid of Bt toxins delayed resistance longer than single-toxin maize, even with a moderate amount of previous resistance to Cry3Bb1 (initial resistance allele frequency = 0.35) but to a lesser extent than when Cry3Bb1 resistance was absent (Fig. 2B).

Increasing the level of resistance to Cry3Bb1 reduced the time to resistance for a pyramid of Cry3Bb1 and Gpp34/Tpp35Ab1, with delays in resistance achieved by the pyramid showing a similar pattern to single-toxin Gpp34/Tpp35Ab1 maize when the initial resistant allele frequency for Cry3Bb1 was 0.8 (Fig. 2C). The time to resistance for the pyramid was always bound between the Cry3Bb1 initial resistance allele frequency of 0.2, representing a pyramid with no previous Cry3Bb1 resistance, and single-toxin Gpp34/Tpp35Ab1 maize (Fig. 2C). The proportion refuge needed to delay resistance by 20 generations for a pyramid with previous Cry3Bb1 resistance ranged from 0.46 to 0.66 when baseline values were used for inheritance of resistance and fitness costs (Fig. 2C).

Lower inheritance of resistance (*h* = 0.14) resulted in longer delays in time to resistance for single-toxin and pyramid Bt maize, in scenario 3, compared to higher inheritance of resistance (*h* = 0.73), in scenario 4 (Fig. 3A and B). Increasing the proportion of the landscape planted to non-Bt refuges increased the number of generations until resistance. In both cases, the delays in resistance were greatest for the pyramid, and smallest for maize with single Bt traits (Fig. 3A and B). When a moderate level of Cry3Bb1 resistance was introduced, delays in the resistance achieved by the Bt pyramid were intermediate between a pyramid without prior resistance and Gpp34/Tpp35Ab1 maize (Fig. 3A and B). Lower inheritance of resistance increased the effectiveness of pyramids to delay resistance, relative to single-toxin Bt maize, compared with higher inheritance of resistance (Fig. 3A and B). For both low and high values of inheritance, we found that increasing the initial frequency of resistance to Cry3Bb1 reduced the capacity of the pyramid to delay resistance (Fig. 3C and D). When the initial resistance allele frequency for Cry3Bb1 resistance was set to 0.8, resistance evolved in a similar time frame for the pyramid and single-toxin Gpp34/Tpp35Ab1 maize (Fig. 3C and D).

Larger, nonrecessive fitness costs delayed resistance compared to the absence of fitness costs (Fig. 4A and B). As with our other simulations, increasing the proportion of refuge increased the number of generations until resistance evolved. General patterns in the time until resistance were similar to other scenarios, with resistance arising in the fewest number of generations for maize with a single Bt toxin, and the greatest delays in resistance arising for maize with a pyramid of Bt toxins (Fig. 4A and B). A moderate level of Cry3Bb1 resistance produced delays in resistance that were intermediate between a pyramid without prior resistance and maize with a single Bt trait. Regardless of whether fitness costs were absent, or set to the largest value, increasing the initial frequency for Cry3Bb1 resistance reduced the capacity of the pyramid to delay resistance. When resistance to Cry3Bb1 was prevalent within a population (i.e., 0.80) the time to resistance was similar between the Bt pyramid and maize with only Gpp34/Tpp35Ab1 (Fig. 4C and D).

## Discussion

We found that the ability of the pyramid of Cry3Bb1 and Gpp34/Tpp35Ab1 to delay resistance was reduced as initial levels of Cry3Bb1 resistance increased. At the highest initial allele frequency for Cry3Bb1 resistance evaluated in this study (0.80), delays in resistance achieved by this Bt pyramid were similar to those observed for single-toxin Gpp34/Tpp35Ab1 maize (Figs. 2C, 3C, 3D, 4C, and 4D). This effect occurred for scenarios with high versus low values for the inheritance of resistance and fitness costs (Figs. 3 and 4). These results align with theoretical work that describes the impact of initial resistance allele frequency on the evolution of resistance to transgenic crops that produce a pyramid of toxins, with the capacity of the pyramid to delay resistance substantially diminished if resistance to one toxin is already present (Roush 1998). We also found that resistance is likely to be delayed for 5 years or less by maize producing a pyramid of Bt toxins with the current minimum refuge requirement of 5%, even in the absence of previous resistance to either Bt toxin in the pyramid (Figs. 3 and 4) (EPA 2023). These results highlight the value of releasing novel pyramids, for which resistance is not already present, and increasing the proportion of the landscape planted to non-Bt refuges (Tabashnik and Gould 2012, Gassmann and Reisig 2023).

As initial levels of resistance to Cry3Bb1 increased, the ability of the pyramid of Cry3Bb1 and Gpp34/Tpp35Ab1 to delay resistance was diminished compared to single-toxin Bt maize (Figs. 2C, 3C, 3D, 4C, and 4D). The individual toxins Cry3Bb1 and Gpp34/Tpp35Ab1 had been commercially available for 6 and 4 years, respectively, and resistance to the Cry3Bb1 toxin was already present in populations of western corn rootworm, when Cry3Bb1 and Gpp34/Tpp35Ab1became commercially available in a pyramid (Gassmann et al. 2011, Tabashnik and Gould 2012, EPA 2020). Our results mirror reports from the field that found resistance to Gpp34/Tpp35Ab1 and the pyramid of Cry3Bb1 with Gpp34/Tpp35Ab1 in 2013 (Gassmann et al. 2016). Our results highlight the expectation of reduced time to resistance for fields that had a longer history of Cry3Bb1 use compared to fields that had limited or no use of Cry3Bb1 prior to deployment of a Bt pyramid. Similar results have been reported in other resistance evolution models, which indicate that initial resistance to one toxin in the pyramid reduces time to resistance for the pyramid (Brévault et al. 2013, Martinez and Caprio 2016).

In contrast, Gryspeirt and Grégoire (2012) found that a pyramid in which resistance alleles to one of the toxins were not rare still successfully delayed resistance, and caused the simulated insect pest population to become extinct. They used high-dose toxins in their model, where each toxin individually killed greater than 99.99% of susceptible individuals, and resulted in recessive inheritance of resistance (US EPA FIFRA SAP 1998, Gryspeirt and Grégoire 2012). This may account for the differences with our results because Bt traits targeting western corn rootworm are not high-dose, and inheritance was nonrecessive (Tabashnik and Gould 2012, Gassmann 2021). These results indicate that when the pyramid toxins are nonhigh dose, the pyramid delays resistance more closely to a single-toxin Bt crop as initial resistance to one of the toxins increases. To produce the longest delays of resistance, therefore, future pyramids should be comprised of independently acting toxins that have not been previously deployed for pest management, resulting in the highest levels possible of pest susceptibility and redundant killing (Gould 1998, Roush 1998, Andow 2008, Brévault et al. 2013, Carrière et al. 2015, Martinez and Caprio 2016, Gassmann 2021, Gassmann and Reisig 2023).

Incorporating a Bt toxin to which insect pest populations currently display resistance into a pyramid is not unique to Bt maize targeting western corn rootworm. Previous selection of *Helicoverpa zea* (Boddie) (Lepidoptera: Noctuidae) on Cry1Ac cotton facilitated resistance to the pyramid of Cry1Ac and Cry2Ab in the United States, despite weak cross-resistance between the two toxins (Brévault et al. 2013, Tabashnik et al. 2013). Previous selection of pink bollworm, *Pectinophora gossypiella* (Saunders) (Lepidoptera: Gelechiidae), on Cry1Ac cotton hastened resistance to the pyramid of Cry1Ac and Cry2Ab in India (Tabashnik et al. 2013, Carrière et al. 2016). In contrast, rapid replacement of single-toxin Cry1Ac cotton with the pyramid of Cry1Ac and Cry2Ab, and planting of large non-Bt refuges, contributed to extended susceptibility of *Helicoverpa armigera* and *Helicoverpa punctigera* to both toxins in Australia (Carrière et al. 2016). These results highlight both the pitfalls, and potential solutions, if single Bt traits are used singly prior to being placed in a pyramid.

Realistic initial resistant allele frequencies of 0.2 for Cry3Bb1 and 0.075 for Gpp34/Tpp35Ab1 resulted in a short-expected time to resistance (2–7 years) for single-toxin and pyramid maize at current minimum refuge requirements and mirrored reports from Iowa (Figs. 2–4). Specifically, resistance to Cry3Bb1 maize was reported after 6 years of commercialization, 8 years after commercialization for Gpp34/Tpp35Ab1, and 4 years for the pyramid of Cry3Bb1 with Gpp34/Tpp35Ab1 (Gassmann et al. 2011, 2016). In contrast, a low initial resistant allele frequency, among other factors including recessive inheritance of resistance, has likely contributed to several decades of successful pest suppression of European corn borer, *Ostrinia nubilalis* (Hübner) (Lepidoptera: Crambidae) with Bt maize in the United States (Hutchison et al. 2010, Smith et al. 2019, Tabashnik et al. 2023a). Our results show that time to resistance for a pyramid is reduced when there is previous resistance to one of the toxins in the pyramid, even when the initial resistance allele frequency to the second toxin is still low (1 in 1,000), and that resistance evolution to the pyramid is further accelerated when the initial resistance allele frequency to the second toxin is higher (7 in 100) (Fig. 2). Elevated initial resistance allele frequencies in western corn rootworm, prior to commercialization of Bt maize targeting this pest, combined with small refuge sizes likely contributed to rapid resistance evolution to single-toxin Bt maize (Onstad and Meinke 2010, Tabashnik and Gould 2012, Gassmann 2021). Our results highlight the expectation that evolution of resistance to Bt traits will be delayed when the proportion of land planted to non-Bt refuges is increased, even if resistance alleles are not rare and previous resistance to one of the toxins in a pyramid is already present (Fig. 2).

Higher inheritance of resistance resulted in fewer generations to resistance compared to lower inheritance of resistance for single-trait and pyramid Bt maize (Fig. 3). Resistance models frequently show that nonrecessive inheritance, including additive inheritance, decreases time to resistance (Gould 1998, Roush 1998, Onstad et al. 2001, Storer 2003, Crowder and Onstad 2005, Tabashnik et al. 2008, Carrière et al. 2010, Tabashnik and Gould 2012). Huang (2021) found that the average inheritance of resistance in cases of field-evolved resistance was 0.63, supporting the expectation of reduced time to resistance when inheritance of resistance is nonrecessive. Interestingly, differences in time to resistance among the pyramids with a range of previous resistance to Cry3Bb1 were smaller when inheritance of resistance was higher (i.e., nonrecessive inheritance) compared to lower inheritance of resistance (i.e., near-recessive inheritance); this suggests that resistance evolution in a pyramid is less sensitive to previous resistance to Cry3Bb1 when inheritance of resistance is higher (Fig. 3C and D). However, it is important to note that resistance always evolved faster at higher versus lower inheritance of resistance (Fig. 3C). As the inheritance of resistance increases, use of larger non-Bt refuges offer one approach to mitigate the increased risk of resistance (Fig. 3) (Roush 1998, Tabashnik et al. 2008).

Fitness costs select against resistance in the refuge and higher nonrecessive fitness costs result in longer delays of resistance compared to the absence of fitness costs (Fig. 4) (Gassmann et al. 2009a). Fitness costs for Gpp34/Tpp35Ab1 resistance may be greater than costs for Cry3Bb1 resistance, although data on fitness costs for field-evolved resistance to Gpp34/Tpp35Ab1 are lacking (Ingber and Gassmann 2015, Paolino and Gassmann 2017, St. Clair et al. 2020, Smith et al. 2023). Resistance was delayed the longest in a pyramid without previous resistance to Cry3Bb1 (Fig. 4C and D). Differences in the time to resistance among simulations were minimal at current minimum refuge sizes (5% for pyramid maize, 10% for single-toxin maize), but even pyramids with previous resistance to Cry3Bb1 delayed resistance longer than single-toxin Bt maize as refuge size increased (Fig. 4) (EPA 2023). These results further highlight the value of increasing refuge sizes to improve resistance management for Bt maize targeting rootworm.

Preparation of this manuscript revealed that a discussion of practical goals for resistance management for Bt crops is often lacking, as noted by Onstad and Knolhoff (2023). For 26 cases, the average time from commercialization to resistance for a Bt crop was 6.5 years, while in another 30 cases susceptibility has been maintained for 2–24 years (Tabashnik et al. 2023b). In conjunction with other management tools, Bt cotton played a major role in the eradication of pink bollworm, *P. gossypiella*, from the United States (Tabashnik et al. 2021). This raises the question: what are the levels of pest suppression or delays in resistance evolution that constitute successful resistance management? Studies often focus on understanding the ecology and evolution of resistance, and this work could be enhanced by framing results in context of practical goals for resistance management that consider economic benefits, desired delays in resistance evolution, and suggested refuge sizes (Crowder and Onstad 2005, Tabashnik and Gould 2012, Sparks 2013, Onstad et al. 2014, NASEM 2016, Gould et al. 2018, Onstad and Knolhoff 2023). More research is needed to address these issues and to formulate practical goals for resistance management.

We found that previous resistance by western corn rootworm to one of the toxins in a pyramid facilitated resistance to the pyramid, a result supported by previous models and confirmed in the field (Brévault et al. 2013, Carrière et al. 2016, Martinez and Caprio 2016). Increased risk of resistance evolution can often be mitigated by implementing larger refuges (Gryspeirt and Grégoire 2012, Brévault et al. 2013, Huang 2021, Tabashnik et al. 2023b). Delays in resistance evolution to future transgenic crops, targeting western corn rootworm or other insect pests, could be increased by combining novel toxins into pyramids, growing these novel pyramids in conjunction with abundant refuges, and using them as part of a broader integrated pest management approach (Tabashnik et al. 2008, Gassmann and Reisig 2023, Gassmann and Meinke 2024).

## Supplementary data

Supplementary data are available at *Journal of Economic Entomology* online.

toae260_suppl_Supplementary_Appendix_1

toae260_suppl_Supplementary_Appendix_2

toae260_suppl_Supplementary_Appendix_3

toae260_suppl_Supplementary_Appendix_4

toae260_suppl_Supplementary_Tables_1
