# Supplementary material for: Applying a computer model to evaluate the evolution of resistance by western corn rootworm to multiple Bt traits in transgenic maize
Source: J Econ Entomol. 2024 Nov 5;117(6):2646–57. doi: 10.1093/jee/toae260 (PMC11682954; doi:10.1093/jee/toae260)
Supplement: toae260_suppl_Supplementary_Appendix_1 [file toae260_suppl_supplementary_appendix_1.pdf]

Appendix 1

General information about the code

Jupyter Notebook can be accessed in the free software bundle ‘Anaconda.’

Text after a pound sign (#) denotes what the code does and tells Jupyter Notebook to ignore any text on that line. Thus, each line in a block of annotation starts with a pound sign (#). The definitions of the parameters are constant across simulations.

Annotations are more detailed in Appendix 2 than in Appendix 3 and 4 since most of the code is the same.

Where there are multiple values for a parameter, those values are listed in brackets with a comma between each value. Example: [3, 6, 9, 10]

Indentation spacing in critical for the program to function properly. If indentations are out of alignment, the code will not run and will produce an error message. Keep trying until it is correct.

<u>Definitions of parameters:</u>
r1_start = starting resistance allele frequency for locus 1, Cry3Bb1
r2_start = starting resistance allele frequency for locus 2, Gpp34/Tpp35Ab1
start_pop = starting population size
refuge = refuge size(s)
gen_fecundity = fecundity of females
winter_mort = winter egg mortality
s1s1_surv_Bt = relative survival of homozygous susceptible for Cry3Bb1 on Cry3Bb1 maize
s1r1_surv_Bt = relative survival of heterozygotes for Cry3Bb1 on Cry3Bb1 maize
r1r1_surv_B t= relative survival of homozygous resistant for Cry3Bb1 on Cry3Bb1
maizes2s2_surv_Bt = relative survival of homozygous susceptible for 34/35 on 34/35 maize
s2r2_surv_Bt = relative survival of heterozygotes for 34/35 on 34/35 maize
r2r2_surv_Bt = relative survival of homozygous resistant for 34/35 on 34/35 maize
s1s1_surv_ref = relative survival of homozygous susceptible for Cry3Bb1 on non-Bt maize
s1r1_surv_ref = relative survival of heterozygotes for Cry3Bb1 on non-Bt maize
r1r1_surv_ref = relative survival of homozygous resistant for Cry3Bb1 on non-Bt maize
s2s2_surv_ref = relative survival of homozygous susceptible for 34/35 on non-Bt maize
s2r2_surv_ref = relative survival of heterozygotes for 34/35 on non-Bt maize
r2r2_surv_ref = relative survival of homozygous resistant for 34/35 on non-Bt maize
