# Supplementary material for: Applying a computer model to evaluate the evolution of resistance by western corn rootworm to multiple Bt traits in transgenic maize
Source: J Econ Entomol. 2024 Nov 5;117(6):2646–57. doi: 10.1093/jee/toae260 (PMC11682954; doi:10.1093/jee/toae260)
Supplement: toae260_suppl_Supplementary_Appendix_2 [file toae260_suppl_supplementary_appendix_2.pdf]

Appendix 2

```
# Code for reproducing Figure 1 of Roush 1998.
# reproducing Roush 1998, Fig. 1; 1st loci neutral

#imports necessary packages for pre-described functions
import math
import matplotlib.pyplot as plt

#defining the name of the function ‘pyramid’ followed by the name of each parameter to
#be entered; no values are entered here. This code only names the function and its
#variables.
def pyramid(r1_start, r2_start, start_pop, refuge, gen_fecundity, winter_mort,
            s1s1_surv_Bt, s1r1_surv_Bt, r1r1_surv_Bt,
            s2s2_surv_Bt, s2r2_surv_Bt, r2r2_surv_Bt,
            s1s1_surv_ref, s1r1_surv_ref, r1r1_surv_ref,
            s2s2_surv_ref, s2r2_surv_ref, r2r2_surv_ref):

    #lists for freq_r, pop_size, t_resist, rs_mort to track data
    freq_r1 = [[] for x in refuge]
    freq_r2 = [[] for x in refuge]
    pop_size = [[] for x in refuge]
    t_resist = [[] for x in refuge]
    s1r1_Bt_mort = [[] for x in refuge]
    s2r2_Bt_mort = [[] for x in refuge]
    last_freq_r1 = [[] for x in refuge]
    last_freq_r2 = [[] for x in refuge]

#iterates through the ranges of parameter values that are being explored, in this case
#refuge size and heterozygote survival on Bt
    for i in range(len(refuge)):
        for j in range(len(s1r1_surv_Bt)):
            Bt = 1 - refuge[i]
            pop_size[i].append(start_pop)
            freq_r1[i].append(r1_start)
            freq_r2[i].append(r2_start)
            t = 0

            #calculating genotype survival in Bt
            s1s1s2s2_surv_Bt = s1s1_surv_Bt * s2s2_surv_Bt
            s1r1s2s2_surv_Bt = s1r1_surv_Bt[j] * s2s2_surv_Bt
            r1r1s2s2_surv_Bt = r1r1_surv_Bt * s2s2_surv_Bt
            s1s1s2r2_surv_Bt = s1s1_surv_Bt * s2r2_surv_Bt[j]
            s1r1s2r2_surv_Bt = s1r1_surv_Bt[j] * s2r2_surv_Bt[j]
            r1r1s2r2_surv_Bt = r1r1_surv_Bt * s2r2_surv_Bt[j]
            s1s1r2r2_surv_Bt = s1s1_surv_Bt * r2r2_surv_Bt
```

```
        s1r1r2r2_surv_Bt = s1r1_surv_Bt[j] * r2r2_surv_Bt
        r1r1r2r2_surv_Bt = r1r1_surv_Bt * r2r2_surv_Bt

        #calculating genotype survival in non-Bt
        s1s1s2s2_surv_ref = s1s1_surv_ref * s2s2_surv_ref
        s1r1s2s2_surv_ref = s1r1_surv_ref * s2s2_surv_ref
        r1r1s2s2_surv_ref = r1r1_surv_ref * s2s2_surv_ref
        s1s1s2r2_surv_ref = s1s1_surv_ref * s2r2_surv_ref
        s1r1s2r2_surv_ref = s1r1_surv_ref * s2r2_surv_ref
        r1r1s2r2_surv_ref = r1r1_surv_ref * s2r2_surv_ref
        s1s1r2r2_surv_ref = s1s1_surv_ref * r2r2_surv_ref
        s1r1r2r2_surv_ref = s1r1_surv_ref * r2r2_surv_ref
        r1r1r2r2_surv_ref = r1r1_surv_ref * r2r2_surv_ref

        for w in range(120):
            #calculating populations size and 's' allele frequencies
            egg_surv = pop_size[i][-1] * (1-winter_mort)
            s1 = 1 - freq_r1[i][-1]
            s2 = 1 - freq_r2[i][-1]

            #Hardy-Weinberg equilibrium genotype calculations
            #s1s1s2s2 = egg_surv * (s1 ** 2) * (s2 ** 2)
            #s1r1s2s2 = egg_surv * (s1 * freq_r1[i][-1] * 2) * (s2 ** 2)
            #r1r1s2s2 = egg_surv * (freq_r1[i][-1] ** 2) * (s2 ** 2)
            #s1s1s2r2 = egg_surv * (s1 ** 2) * (s2 * freq_r2[i][-1] * 2)
            #s1r1s2r2 = egg_surv * (s1 * freq_r1[i][-1] * 2) * (s2 * freq_r2[i][-1] * 2)
            #r1r1s2r2 = egg_surv * (freq_r1[i][-1] ** 2) * (s2 * freq_r2[i][-1] * 2)
            #s1s1r2r2 = egg_surv * (s1 ** 2) * (freq_r2[i][-1] ** 2)
            #s1r1r2r2 = egg_surv * (s1 * freq_r1[i][-1] * 2) * (freq_r2[i][-1] ** 2)
            #r1r1r2r2 = egg_surv * (freq_r1[i][-1] ** 2) * (freq_r2[i][-1] ** 2)

            #Bt selection
            adult_s1s1s2s2_Bt = egg_surv * Bt * (s1 ** 2) * (s2 ** 2) * s1s1s2s2_surv_Bt
            adult_s1r1s2s2_Bt = egg_surv * Bt * (s1 * freq_r1[i][-1] * 2) * (s2 ** 2) *
                        s1r1s2s2_surv_Bt
            adult_r1r1s2s2_Bt = egg_surv * Bt * (freq_r1[i][-1] ** 2) * (s2 ** 2) *
                        r1r1s2s2_surv_Bt
            adult_s1s1s2r2_Bt = egg_surv * Bt * (s1 ** 2) * (s2 * freq_r2[i][-1] * 2) *
                       s1s1s2r2_surv_Bt
            adult_s1r1s2r2_Bt = egg_surv * Bt * (s1 * freq_r1[i][-1] * 2) * (s2 * freq_r2[i][-1]
                        * 2) * s1r1s2r2_surv_Bt
            adult_r1r1s2r2_Bt = egg_surv * Bt * (freq_r1[i][-1] ** 2) * (s2 * freq_r2[i][-1] * 2)
                        * r1r1s2r2_surv_Bt
            adult_s1s1r2r2_Bt = egg_surv * Bt * (s1 ** 2) * (freq_r2[i][-1] ** 2) *
                        s1s1r2r2_surv_Bt
```

```
adult_s1r1r2r2_Bt = egg_surv * Bt * (s1 * freq_r1[i][-1] * 2) * (freq_r2[i][-1] ** 2)
        * s1r1r2r2_surv_Bt
adult_r1r1r2r2_Bt = egg_surv * Bt * (freq_r1[i][-1] ** 2) * (freq_r2[i][-1] ** 2) *
        r1r1r2r2_surv_Bt

adults_Bt = (adult_s1s1s2s2_Bt + adult_s1r1s2s2_Bt + adult_r1r1s2s2_Bt +
        adult_s1s1s2r2_Bt + adult_s1r1s2r2_Bt + adult_r1r1s2r2_Bt +
        adult_s1s1r2r2_Bt + adult_s1r1r2r2_Bt + adult_r1r1r2r2_Bt)

#refuge[i] survival
adult_s1s1s2s2_ref = egg_surv * refuge[i] * (s1 ** 2) * (s2 ** 2) *
        s1s1s2s2_surv_ref
adult_s1r1s2s2_ref = egg_surv * refuge[i] * (s1 * freq_r1[i][-1] * 2) * (s2 ** 2) *
        s1r1s2s2_surv_ref
adult_r1r1s2s2_ref = egg_surv * refuge[i] * (freq_r1[i][-1] ** 2) * (s2 ** 2) *
        r1r1s2s2_surv_ref
adult_s1s1s2r2_ref = egg_surv * refuge[i] * (s1 ** 2) * (s2 * freq_r2[i][-1] * 2) *
        s1s1s2r2_surv_ref
adult_s1r1s2r2_ref = egg_surv * refuge[i] * (s1 * freq_r1[i][-1] * 2) * (s2 *
        freq_r2[i][-1] * 2) * s1r1s2r2_surv_ref
adult_r1r1s2r2_ref = egg_surv * refuge[i] * (freq_r1[i][-1] ** 2) * (s2 *
        freq_r2[i][-1] * 2) * r1r1s2r2_surv_ref
adult_s1s1r2r2_ref = egg_surv * refuge[i] * (s1 ** 2) * (freq_r2[i][-1] ** 2) *
        s1s1r2r2_surv_ref
adult_s1r1r2r2_ref = egg_surv * refuge[i] * (s1 * freq_r1[i][-1] * 2) * (freq_r2[i][-
        1] ** 2) * s1r1r2r2_surv_ref
adult_r1r1r2r2_ref = egg_surv * refuge[i] * (freq_r1[i][-1] ** 2) * (freq_r2[i][-1] **
        2) * r1r1r2r2_surv_ref

adults_ref = (adult_s1s1s2s2_ref + adult_s1r1s2s2_ref + adult_r1r1s2s2_ref +
        adult_s1s1s2r2_ref + adult_s1r1s2r2_ref + adult_r1r1s2r2_ref +
        adult_s1s1r2r2_ref + adult_s1r1r2r2_ref + adult_r1r1r2r2_ref)

#Calculations of allele frequencies (these are used to calculate the
#proportions of alleles contributed to the next gen by surviving adults
Bt_r1_nextgen = ((adult_s1r1s2s2_Bt) + (adult_s1r1s2r2_Bt) +
        (adult_s1r1r2r2_Bt) + 2 * ((adult_r1r1s2s2_Bt ) + (adult_r1r1s2r2_Bt)
        + (adult_r1r1r2r2_Bt)))
Bt_r2_nextgen = ((adult_s1s1s2r2_Bt) + (adult_s1r1s2r2_Bt) +
        (adult_r1r1s2r2_Bt) + 2 * ((adult_s1s1r2r2_Bt) + (adult_s1r1r2r2_Bt)
        + (adult_r1r1r2r2_Bt)))
Bt_s1_nextgen = ((adult_s1r1s2s2_Bt) + (adult_s1r1s2r2_Bt) +
        (adult_s1r1r2r2_Bt) + 2 * ((adult_s1s1s2s2_Bt) +
        (adult_s1s1s2r2_Bt) + (adult_s1s1r2r2_Bt)))
```

```
        Bt_s2_nextgen = ((adult_s1s1s2r2_Bt) + (adult_s1r1s2r2_Bt) +
                (adult_r1r1s2r2_Bt) + 2 * ((adult_s1s1s2s2_Bt) +
                (adult_s1r1s2s2_Bt) + (adult_r1r1s2s2_Bt)))

        ref_r1_nextgen = ((adult_s1r1s2s2_ref) + (adult_s1r1s2r2_ref) +
                (adult_s1r1r2r2_ref) + 2 * ((adult_r1r1s2s2_ref) +
                (adult_r1r1s2r2_ref) + (adult_r1r1r2r2_ref)))
        ref_r2_nextgen = ((adult_s1s1s2r2_ref) + (adult_s1r1s2r2_ref) +
                (adult_r1r1s2r2_ref) + 2 * ((adult_s1s1r2r2_ref) +
                (adult_s1r1r2r2_ref) + (adult_r1r1r2r2_ref)))
        ref_s1_nextgen = ((adult_s1r1s2s2_ref) + (adult_s1r1s2r2_ref) +
                (adult_s1r1r2r2_ref) + 2  * ((adult_s1s1s2s2_ref) +
                (adult_s1s1s2r2_ref) + (adult_s1s1r2r2_ref)))
        ref_s2_nextgen = ((adult_s1s1s2r2_ref) + (adult_s1r1s2r2_ref) +
                (adult_r1r1s2r2_ref)+ 2  * ((adult_s1s1s2s2_ref) +
                (adult_s1r1s2s2_ref) + (adult_r1r1s2s2_ref)))

        #calculates new frequencies
        new_freq_r1 = (Bt_r1_nextgen + ref_r1_nextgen) / (Bt_r1_nextgen +
                ref_r1_nextgen + Bt_s1_nextgen + ref_s1_nextgen)
        new_freq_r2 = (Bt_r2_nextgen + ref_r2_nextgen) / (Bt_r2_nextgen +
                ref_r2_nextgen + Bt_s2_nextgen + ref_s2_nextgen)

        freq_r1[i].append(new_freq_r1)
        freq_r2[i].append(new_freq_r2)

        #calculations of F(t+1) egg population size; Bt/ref denotes eggs produced by
        #adults surviving in each corn type 'gen_fecundity/2' because, assuming
        #50:50 M:F, only half of the individuals (the females) will produce eggs
        eggs_Bt = ((gen_fecundity / 2) *
                ((adult_s1s1s2s2_Bt) + (adult_s1r1s2s2_Bt) + (adult_r1r1s2s2_Bt) +
                (adult_s1s1s2r2_Bt) + (adult_s1r1s2r2_Bt) + (adult_r1r1s2r2_Bt) +
                (adult_s1s1r2r2_Bt) + (adult_s1r1r2r2_Bt) + (adult_r1r1r2r2_Bt)))

        eggs_ref = ((gen_fecundity / 2) *
                ((adult_s1s1s2s2_ref) + (adult_s1r1s2s2_ref) + (adult_r1r1s2s2_ref) +
                (adult_s1s1s2r2_ref) + (adult_s1r1s2r2_ref) + (adult_r1r1s2r2_ref) +
                (adult_s1s1r2r2_ref) + (adult_s1r1r2r2_ref) + (adult_r1r1r2r2_ref)))

        next_gen = eggs_Bt + eggs_ref
        pop_size.append(next_gen)

        # t is a counter to keep track of generations
        t = t + 1
```

```
                # conditional operation to check the frequency of resistance alleles and track
                # them based on either locus exceeding 50%
                if  new_freq_r1 > 0.5 or new_freq_r2 > 0.5:
                    t_resist[i].append(t)
                    s1r1_Bt_mort[i].append(1 - s1r1_surv_Bt[j])
                    s2r2_Bt_mort[i].append(1 - s2r2_surv_Bt[j])
                    last_freq_r1[i].append(freq_r1[i][-1])
                    last_freq_r2[i].append(freq_r2[i][-1])
                    break

    # graphing results
    for k in range(len(t_resist)):
        plt.plot(s2r2_Bt_mort[k], t_resist[k], label = 'Refuge: ' + str(refuge[k] * 100) + '%')
        plt.legend(loc = 'upper left')
        plt.title('Neutral 1st Locus; repr. Fig 1 Roush 1998')
        plt.xlim(0, 1)
        plt.ylim(0, 100)
        plt.xlabel('Mortality of Heterozygotes')
        plt.ylabel('Generations Until [R] > 50%')
        plt.show

    #return (last_freq_r1, last_freq_r2, s1r1_Bt_mort,s2r2_Bt_mort, t_resist)

#enter parameter values here, in the order listed below. s1r1_surv_Bt must have the
#same number #of values in [ ] as s2r2_surv_Bt. Values at the nth position in
#s1r1_surv_Bt and s2r2_surv_Bt will be used in simulation n.
#Length of refuge list does not have to be the same length as heterozygote survival.
#r1_start, r2_start, start_pop, ref, gen_fecundity, winter_mort,
#s1s1_surv_Bt, s1r1_surv_Bt, r1r1_surv_Bt,
#s2s2_surv_Bt, s2r2_surv_Bt, r2r2_surv_Bt,
#s1s1_surv_ref, s1r1_surv_ref, r1r1_surv_ref,
#s2s2_surv_ref, s2r2_surv_ref, r2r2_surv_ref,

pyramid(0.001, 0.001, 10000, [0.1, 0.2, 0.5], 350, 0.95,
        1, [1, 1, 1, 1, 1, 1, 1, 1, 1, 1, 1, 1, 1], 1,
        0, [1, 0.5, 0.4, 0.3, 0.2, 0.1, 0.075, 0.05, 0.04, 0.03, 0.02, 0.01, 0], 1,
        1, 1, 1,
        1, 1, 1)
```
