# Supplementary material for: Applying a computer model to evaluate the evolution of resistance by western corn rootworm to multiple Bt traits in transgenic maize
Source: J Econ Entomol. 2024 Nov 5;117(6):2646–57. doi: 10.1093/jee/toae260 (PMC11682954; doi:10.1093/jee/toae260)
Supplement: toae260_suppl_Supplementary_Appendix_3 [file toae260_suppl_supplementary_appendix_3.pdf]

# Appendix 3

```
#Example of code for single-toxin Bt maize
# Single-toxin 3Bb1 code for all simulations

import time
import math
import matplotlib.pyplot as plt
import pandas as pd

#defining the name of the function 'pyramid' followed by the name of each parameter to
#be entered; no values are entered here. This code only names the function and its
#variables.
def pyramid(r1_start, r2_start, start_pop, refuge, gen_fecundity, winter_mort,
            s1s1_surv_Bt, s1r1_surv_Bt, r1r1_surv_Bt,
            s2s2_surv_Bt, s2r2_surv_Bt, r2r2_surv_Bt,
            s1s1_surv_ref, s1r1_surv_ref, r1r1_surv_ref,
            s2s2_surv_ref, s2r2_surv_ref, r2r2_surv_ref):

    start = time.time()
    #lists for freq_r, pop_size, t_resist, rs_mort
    freq_r1 = [[[] for y in refuge] for x in s1r1_surv_Bt]
    freq_r2 = [[[] for y in refuge] for x in s1r1_surv_Bt]
    pop_size = [[] for x in s1r1_surv_Bt]
    t_resist = [[] for x in s1r1_surv_Bt]
    s1r1_Bt_mort = [[] for x in s1r1_surv_Bt]
    s2r2_Bt_mort = [[] for x in s1r1_surv_Bt]
    last_freq_r1 = [[] for x in s1r1_surv_Bt]
    last_freq_r2 = [[] for x in s1r1_surv_Bt]
    ref_size = [[] for x in s1r1_surv_Bt]
    for_excel = []

    simulation = ['2A', '2B', '3A', '3C', '4A', '4C']
    #line_style = ['solid', 'dashed', 'dashdot', 'dotted']
    #colors = ['darkorange', 'purple', 'royalblue', 'black']

    #iterates through the ranges of parameter values that are being explored, in this case
    #refuge size and heterozygote survival on Bt
    for j in range(len(s1r1_surv_Bt)):
        for i in range(len(refuge)):

            Bt = 1 - refuge[i]
            pop_size[j].append(start_pop)
            freq_r1[j][i].append(r1_start[j])
            freq_r2[j][i].append(r2_start[j])
            t = 0
```

```
    #calculating genotype survival in Bt
    s1s1s2s2_surv_Bt = s1s1_surv_Bt * s2s2_surv_Bt
    s1r1s2s2_surv_Bt = s1r1_surv_Bt[j] * s2s2_surv_Bt
    r1r1s2s2_surv_Bt = r1r1_surv_Bt[j] * s2s2_surv_Bt
    s1s1s2r2_surv_Bt = s1s1_surv_Bt * s2r2_surv_Bt[j]
    s1r1s2r2_surv_Bt = s1r1_surv_Bt[j] * s2r2_surv_Bt[j]
    r1r1s2r2_surv_Bt = r1r1_surv_Bt[j] * s2r2_surv_Bt[j]
    s1s1r2r2_surv_Bt = s1s1_surv_Bt * r2r2_surv_Bt[j]
    s1r1r2r2_surv_Bt = s1r1_surv_Bt[j] * r2r2_surv_Bt[j]
    r1r1r2r2_surv_Bt = r1r1_surv_Bt[j] * r2r2_surv_Bt[j]

    #calculating genotype survival in non-Bt
    s1s1s2s2_surv_ref = s1s1_surv_ref * s2s2_surv_ref
    s1r1s2s2_surv_ref = s1r1_surv_ref[j] * s2s2_surv_ref
    r1r1s2s2_surv_ref = r1r1_surv_ref[j] * s2s2_surv_ref
    s1s1s2r2_surv_ref = s1s1_surv_ref * s2r2_surv_ref[j]
    s1r1s2r2_surv_ref = s1r1_surv_ref[j] * s2r2_surv_ref[j]
    r1r1s2r2_surv_ref = r1r1_surv_ref[j] * s2r2_surv_ref[j]
    s1s1r2r2_surv_ref = s1s1_surv_ref * r2r2_surv_ref[j]
    s1r1r2r2_surv_ref = s1r1_surv_ref[j] * r2r2_surv_ref[j]
    r1r1r2r2_surv_ref = r1r1_surv_ref[j] * r2r2_surv_ref[j]

    for w in range(120):
        #calculating populations size and 's' allele frequencies
        egg_surv = pop_size[j][-1] * (1-winter_mort)
        s1 = 1 - freq_r1[j][i][-1]
        s2 = 1 - freq_r2[j][i][-1]
        r1 = 1 - s1
        r2 = 1 - s2

        #Hardy-Weinberg equilibrium genotype calculations
        #s1s1s2s2 = egg_surv * (s1 ** 2) * (s2 ** 2)
        #s1r1s2s2 = egg_surv * (s1 * freq_r1[j][-1] * 2) * (s2 ** 2)
        #r1r1s2s2 = egg_surv * (freq_r1[j][-1] ** 2) * (s2 ** 2)
        #s1s1s2r2 = egg_surv * (s1 ** 2) * (s2 * freq_r2[j][-1] * 2)
        #s1r1s2r2 = egg_surv * (s1 * freq_r1[j][-1] * 2) * (s2 * freq_r2[j][-1] * 2)
        #r1r1s2r2 = egg_surv * (freq_r1[j][-1] ** 2) * (s2 * freq_r2[j][-1] * 2)
        #s1s1r2r2 = egg_surv * (s1 ** 2) * (freq_r2[j][-1] ** 2)
        #s1r1r2r2 = egg_surv * (s1 * freq_r1[j][-1] * 2) * (freq_r2[j][-1] ** 2)
        #r1r1r2r2 = egg_surv * (freq_r1[j][-1] ** 2) * (freq_r2[j][-1] ** 2)

        #Bt selection
        adult_s1s1s2s2_Bt = egg_surv * Bt * (s1 ** 2) * (s2 ** 2) * s1s1s2s2_surv_Bt
                adult_s1r1s2s2_Bt = egg_surv * Bt * (s1 * r1 * 2) * (s2 ** 2) *
                s1r1s2s2_surv_Bt
```

```
        adult_r1r1s2s2_Bt = egg_surv * Bt * (r1 ** 2) * (s2 ** 2) * r1r1s2s2_surv_Bt
        adult_s1s1s2r2_Bt = egg_surv * Bt * (s1 ** 2) * (s2 * r2 * 2) *
                s1s1s2r2_surv_Bt
        adult_s1r1s2r2_Bt = egg_surv * Bt * (s1 * r1 * 2) * (s2 * r2 * 2) *
                s1r1s2r2_surv_Bt
        adult_r1r1s2r2_Bt = egg_surv * Bt * (r1 ** 2) * (s2 * r2 * 2) * r1r1s2r2_surv_Bt
        adult_s1s1r2r2_Bt = egg_surv * Bt * (s1 ** 2) * (r2 ** 2) * s1s1r2r2_surv_Bt
        adult_s1r1r2r2_Bt = egg_surv * Bt * (s1 * r1 * 2) * (r2 ** 2) * s1r1r2r2_surv_Bt
        adult_r1r1r2r2_Bt = egg_surv * Bt * (r1 ** 2) * (r2 ** 2) * r1r1r2r2_surv_Bt

        #refuge[i] survival
        adult_s1s1s2s2_ref = egg_surv * refuge[i] * (s1 ** 2) * (s2 ** 2) *
                s1s1s2s2_surv_ref
        adult_s1r1s2s2_ref = egg_surv * refuge[i] * (s1 * r1 * 2) * (s2 ** 2) *
                s1r1s2s2_surv_ref
        adult_r1r1s2s2_ref = egg_surv * refuge[i] * (r1 ** 2) * (s2 ** 2) *
                r1r1s2s2_surv_ref
        adult_s1s1s2r2_ref = egg_surv * refuge[i] * (s1 ** 2) * (s2 * r2 * 2) *
                s1s1s2r2_surv_ref
        adult_s1r1s2r2_ref = egg_surv * refuge[i] * (s1 * r1 * 2) * (s2 * r2 * 2) *
                s1r1s2r2_surv_ref
        adult_r1r1s2r2_ref = egg_surv * refuge[i] * (r1 ** 2) * (s2 * r2 * 2) *
                r1r1s2r2_surv_ref
        adult_s1s1r2r2_ref = egg_surv * refuge[i] * (s1 ** 2) * (r2 ** 2) *
                s1s1r2r2_surv_ref
        adult_s1r1r2r2_ref = egg_surv * refuge[i] * (s1 * r1 * 2) * (r2 ** 2) *
                s1r1r2r2_surv_ref
        adult_r1r1r2r2_ref = egg_surv * refuge[i] * (r1 ** 2) * (r2 ** 2) *
                r1r1r2r2_surv_ref

        adults_Bt = (adult_s1s1s2s2_Bt + adult_s1r1s2s2_Bt + adult_r1r1s2s2_Bt +
                     adult_s1s1s2r2_Bt + adult_s1r1s2r2_Bt + adult_r1r1s2r2_Bt +
                     adult_s1s1r2r2_Bt + adult_s1r1r2r2_Bt + adult_r1r1r2r2_Bt)

        adults_ref = (adult_s1s1s2s2_ref + adult_s1r1s2s2_ref + adult_r1r1s2s2_ref +
                      adult_s1s1s2r2_ref + adult_s1r1s2r2_ref + adult_r1r1s2r2_ref +
                      adult_s1s1r2r2_ref + adult_s1r1r2r2_ref + adult_r1r1r2r2_ref)

        #Calculations of allele requencies (these are used to calculate the proportions
        #of alleles
        #contributed to the next gen by surviving adults)
        Bt_r1_nextgen = ((adult_s1r1s2s2_Bt) + (adult_s1r1s2r2_Bt) +
                (adult_s1r1r2r2_Bt) +
                2 * ((adult_r1r1s2s2_Bt) + (adult_r1r1s2r2_Bt) + (adult_r1r1r2r2_Bt)))
        Bt_r2_nextgen = ((adult_s1s1s2r2_Bt) + (adult_s1r1s2r2_Bt) +
                (adult_r1r1s2r2_Bt) +
```

```
            2 * ((adult_s1s1r2r2_Bt) + (adult_s1r1r2r2_Bt) + (adult_r1r1r2r2_Bt)))
    Bt_s1_nextgen = ((adult_s1r1s2s2_Bt) + (adult_s1r1s2r2_Bt) +
            (adult_s1r1r2r2_Bt) +
            2 * ((adult_s1s1s2s2_Bt) + (adult_s1s1s2r2_Bt) +
            (adult_s1s1r2r2_Bt)))
    Bt_s2_nextgen = ((adult_s1s1s2r2_Bt) + (adult_s1r1s2r2_Bt) +
            (adult_r1r1s2r2_Bt) +
            2 * ((adult_s1s1s2s2_Bt) + (adult_s1r1s2s2_Bt) +
            (adult_r1r1s2s2_Bt)))

    ref_r1_nextgen = ((adult_s1r1s2s2_ref) + (adult_s1r1s2r2_ref) +
            (adult_s1r1r2r2_ref) +
            2 * ((adult_r1r1s2s2_ref) + (adult_r1r1s2r2_ref) + (adult_r1r1r2r2_ref)))
    ref_r2_nextgen = ((adult_s1s1s2r2_ref) + (adult_s1r1s2r2_ref) +
            (adult_r1r1s2r2_ref) +
            2 * ((adult_s1s1r2r2_ref) + (adult_s1r1r2r2_ref) + (adult_r1r1r2r2_ref)))
    ref_s1_nextgen = ((adult_s1r1s2s2_ref) + (adult_s1r1s2r2_ref) +
            (adult_s1r1r2r2_ref) +
            2 * ((adult_s1s1s2s2_ref) + (adult_s1s1s2r2_ref) +
            (adult_s1s1r2r2_ref)))
    ref_s2_nextgen = ((adult_s1s1s2r2_ref) + (adult_s1r1s2r2_ref) +
            (adult_r1r1s2r2_ref)+
            2 * ((adult_s1s1s2s2_ref) + (adult_s1r1s2s2_ref) +
            (adult_r1r1s2s2_ref)))

    new_freq_r1 = (Bt_r1_nextgen + ref_r1_nextgen) / (Bt_r1_nextgen +
            ref_r1_nextgen + Bt_s1_nextgen + ref_s1_nextgen)
    new_freq_r2 = (Bt_r2_nextgen + ref_r2_nextgen) / (Bt_r2_nextgen +
            ref_r2_nextgen + Bt_s2_nextgen + ref_s2_nextgen)

    freq_r1[j][i].append(new_freq_r1)
    freq_r2[j][i].append(new_freq_r2)

    #calculations of F(t+1) egg population size; Bt/ref denotes eggs produced by
    #adults surviving in each corn type 'gen_fecundity/2' because, assuming
    #50:50 M:F, only half of the individuals (the females) will produce eggs
    eggs_Bt = ((gen_fecundity / 2) *
             ((adult_s1s1s2s2_Bt) + (adult_s1r1s2s2_Bt) + (adult_r1r1s2s2_Bt) +
             (adult_s1s1s2r2_Bt) + (adult_s1r1s2r2_Bt) + (adult_r1r1s2r2_Bt) +
             (adult_s1s1r2r2_Bt) + (adult_s1r1r2r2_Bt) + (adult_r1r1r2r2_Bt)))

    eggs_ref = ((gen_fecundity / 2) *
             ((adult_s1s1s2s2_ref) + (adult_s1r1s2s2_ref) + (adult_r1r1s2s2_ref) +
             (adult_s1s1s2r2_ref) + (adult_s1r1s2r2_ref) + (adult_r1r1s2r2_ref) +
             (adult_s1s1r2r2_ref) + (adult_s1r1r2r2_ref) + (adult_r1r1r2r2_ref)))
```

```
            next_gen = eggs_Bt + eggs_ref
            pop_size.append(next_gen)
            t = t + 1

            # conditional operation to check the frequency of resistance alleles and track
            # them based on both loci exceeding 50%
            if  freq_r1[j][i][-1] > 0.5 or freq_r2[j][i][-1] > 0.5:
                t_resist[j].append(t)
                ref_size[j].append(refuge[i])
                s1r1_Bt_mort[j].append(1 - s1r1_surv_Bt[j])
                s2r2_Bt_mort[j].append(1 - s2r2_surv_Bt[j])
                last_freq_r1[j].append(freq_r1[j][-1])
                last_freq_r2[j].append(freq_r2[j][-1])
                b = [refuge[i], t, simulation[j], s1r1_surv_Bt[j], r1r1_surv_ref[j], r1_start[j]]
                for_excel.append(b)
                break
#generates a dataframe for export to excel
results = pd.DataFrame(for_excel)
    results.columns = ['refuge', 'gen_to_resist', 'simulation', 'sr_surv_Bt', 'rr_surv_ref',
         'r1_start']

#plots results
for k in range(len(t_resist)):
    plt.plot(ref_size[k], t_resist[k], label = simulation[k], linewidth = 3,  )
    plt.legend(loc = 'lower right', fontsize = 12)
    #plt.title('Pyramid IRM model', fontsize = 30)
    plt.xlim(0, 1)
    plt.ylim(0, 20)
    plt.xticks(fontsize = 16)
    plt.yticks(fontsize = 16)
    plt.xlabel('Proportion refuge', fontsize = 22)
    plt.ylabel('Generations Until [R] > 50%', fontsize = 22)
    plt.show

end = time.time()
runtime = end - start

#exports data to excel. Enter the EXACT filepath and name, ending in .xlsx
results.to_excel(r'insert file path and name here.xlsx', sheet_name = '3Bb1')

#Prints results, or a portion of the results if the dataframe is large
return(results)
#return (t_resist, ref_size, freq_r1[0][0], freq_r2[0][0])
#return (runtime, t_resist, ref_size, last_freq_r1, last_freq_r2)
#return(runtime, s1r1_Bt_mort, s2r2_Bt_mort, last_freq_r1, last_freq_r2)
```

```
#enter parameter values here, in the order listed below. Length of lists for survival must
#be the same length. Values at the nth position in survival parameters will be used in
#simulation n.
#Length of refuge list does not have to be the same length as lists for survival.
#r1_start, r2_start, start_pop, refuge, gen_fecundity, winter_mort,
#s1s1_surv_Bt, s1r1_surv_Bt, r1r1_surv_Bt,(3Bb1 allele)
#s2s2_surv_Bt, s2r2_surv_Bt, r2r2_surv_Bt, (34/35 allele)
#s1s1_surv_ref, s1r1_surv_ref, r1r1_surv_ref,(3Bb1 allele)
#s2s2_surv_ref, s2r2_surv_ref, r2r2_surv_ref,(34/35 allele)

pyramid([0.001, 0.2, 0.2, 0.2, 0.2, 0.2], [0.05, 0.05, 0.05, 0.05, 0.05, 0.05], 100000,
    [0.00, 0.01, 0.02, 0.03, 0.04, 0.05, 0.06, 0.07, 0.08, 0.09, 0.10,
    0.11, 0.12, 0.13, 0.14, 0.15, 0.16, 0.17, 0.18, 0.19, 0.20,
    0.21, 0.22, 0.23, 0.24, 0.25, 0.26, 0.27, 0.28, 0.29, 0.30,
    0.31, 0.32, 0.33, 0.34, 0.35, 0.36, 0.37, 0.38, 0.39, 0.40,
    0.41, 0.42, 0.43, 0.44, 0.45, 0.46, 0.47, 0.48, 0.49, 0.50,
    0.51, 0.52, 0.53, 0.54, 0.55, 0.56, 0.57, 0.58, 0.59, 0.60,
    0.61, 0.62, 0.63, 0.64, 0.65, 0.66, 0.67, 0.68, 0.69, 0.70,
    0.71, 0.72, 0.73, 0.74, 0.75, 0.76, 0.77, 0.78, 0.79, 0.80,
    0.81, 0.82, 0.83, 0.84, 0.85, 0.86, 0.87, 0.88, 0.89, 0.90,
    0.91, 0.92, 0.93, 0.94, 0.95, 0.96, 0.97, 0.98, 0.99, 1.00], 350, 0.95,
    0.104, [0.44, 0.44, 0.229, 0.758, 0.44, 0.44], [1, 1, 1, 1, 1, 1],
    1, [1, 1, 1, 1, 1, 1], [1, 1, 1, 1, 1, 1],
    1, [1, 1, 1, 1, 1, 0.94], [0.959, 0.959, 0.959, 0.959, 1, 0.85],
    1, [1, 1, 1, 1, 1, 1], [1, 1, 1, 1, 1, 1])
```
