# Supplementary material for: Applying a computer model to evaluate the evolution of resistance by western corn rootworm to multiple Bt traits in transgenic maize
Source: J Econ Entomol. 2024 Nov 5;117(6):2646–57. doi: 10.1093/jee/toae260 (PMC11682954; doi:10.1093/jee/toae260)
Supplement: toae260_suppl_Supplementary_Tables_1 [file toae260_suppl_supplementary_tables_1.docx]

**Supplemental Table 1. Survival for each combination of genotype by maize, among the six scenarios, at the start of a simulation.**

|  | **Scenario 1** | | **Scenario 2** | | **Scenario 3** | | **Scenario 4** | | **Scenario 5** | | **Scenario 6** | |
| --- | --- | --- | --- | --- | --- | --- | --- | --- | --- | --- | --- | --- |
| **Genotype** | **Cry3Bb1** | **non-Bt** | **Cry3Bb1** | **non-Bt** | **Cry3Bb1** | **non-Bt** | **Cry3Bb1** | **non-Bt** | **Cry3Bb1** | **non-Bt** | **Cry3Bb1** | **non-Bt** |
| SS | 0.104 | 1.000 | 0.104 | 1.000 | 0.104 | 1.000 | 0.104 | 1.000 | 0.104 | 1.000 | 0.104 | 1.000 |
| SR | 0.440 | 1.000 | 0.440 | 1.000 | 0.229 | 1.000 | 0.758 | 1.000 | 0.440 | 1.000 | 0.440 | 0.940 |
| RR | 1.000 | 0.959 | 1.000 | 0.959 | 1.000 | 0.959 | 1.000 | 0.959 | 1.000 | 1.000 | 1.000 | 0.850 |
| **Genotype** | **34/35** | **non-Bt** | **34/35** | **non-Bt** | **34/35** | **non-Bt** | **34/35** | **non-Bt** | **34/35** | **non-Bt** | **34/35** | **non-Bt** |
| SS | 0.118 | 1.000 | 0.118 | 1.000 | 0.118 | 1.000 | 0.118 | 1.000 | 0.118 | 1.000 | 0.118 | 1.000 |
| SR | 0.436 | 1.000 | 0.436 | 1.000 | 0.241 | 1.000 | 0.762 | 1.000 | 0.436 | 1.000 | 0.436 | 0.940 |
| RR | 1.000 | 0.945 | 1.000 | 0.945 | 1.000 | 0.945 | 1.000 | 0.945 | 1.000 | 1.000 | 1.000 | 0.850 |
| **Genotype^†^**  **3Bb1-34/35** | **Pyramid** | **non-Bt** | **Pyramid** | **non-Bt** | **Pyramid** | **non-Bt** | **Pyramid** | **non-Bt** | **Pyramid** | **non-Bt** | **Pyramid** | **non-Bt** |
| SS-SS | 0.012 | 1.000 | 0.012 | 1.000 | 0.012 | 1.000 | 0.012 | 1.000 | 0.012 | 1.000 | 0.012 | 1.000 |
| SS-SR | 0.045 | 1.000 | 0.192 | 1.000 | 0.025 | 1.000 | 0.079 | 1.000 | 0.045 | 1.000 | 0.045 | 0.940 |
| SS-RR | 0.104 | 0.945 | 1.000 | 0.945 | 0.104 | 0.945 | 0.104 | 0.945 | 0.104 | 1.000 | 0.104 | 0.850 |
| SR-SS | 0.052 | 1.000 | 0.052 | 1.000 | 0.027 | 1.000 | 0.089 | 1.000 | 0.052 | 1.000 | 0.052 | 0.940 |
| SR-SR | 0.192 | 1.000 | 0.192 | 1.000 | 0.055 | 1.000 | 0.578 | 1.000 | 0.192 | 1.000 | 0.192 | 0.884 |
| SR-RR | 0.440 | 0.945 | 0.440 | 0.945 | 0.229 | 0.945 | 0.758 | 0.945 | 0.440 | 1.000 | 0.440 | 0.799 |
| RR-SS | 0.118 | 0.959 | 0.118 | 0.959 | 0.118 | 0.959 | 0.118 | 0.959 | 0.118 | 1.000 | 0.118 | 0.850 |
| RR-SR | 0.436 | 0.959 | 0.436 | 0.959 | 0.241 | 0.959 | 0.762 | 0.959 | 0.436 | 1.000 | 0.436 | 0.799 |
| RR-RR | 1.000 | 0.906 | 1.000 | 0.906 | 1.000 | 0.906 | 1.000 | 0.906 | 1.000 | 1.000 | 1.000 | 0.723 |

^†^ For the two locus model, the locus for Cry3Bb1 resistance is described first (3Bb1) followed by the locus for Gpp34/Tpp35Ab1 resistance (34/35)
